# Investigation of Marine-Derived Fungal Diversity and Their Exploitable Biological Activities

**DOI:** 10.3390/md13074137

**Published:** 2015-06-30

**Authors:** Joo-Hyun Hong, Seokyoon Jang, Young Mok Heo, Mihee Min, Hwanhwi Lee, Young Min Lee, Hanbyul Lee, Jae-Jin Kim

**Affiliations:** Division of Environmental Science & Ecological Engineering, College of Life Sciences & Biotechnology, Korea University, 145 Anam-ro, Seongbuk-gu, Seoul 136-713, Korea; E-Mails: dress8@korea.ac.kr (J.-H.H.); skel@korea.ac.kr (S.J.); hym011@korea.ac.kr (Y.M.H.); mihee1220@korea.ac.kr (M.M.); ghksgnl27@korea.ac.kr (H.L.); ymlee25@korea.ac.kr (Y.M.L.); hblee95@korea.ac.kr (H.L.)

**Keywords:** antioxidant activity, biological control, cellulolytic enzyme activity, marine fungi, phylogenetic analysis

## Abstract

Marine fungi are potential producers of bioactive compounds that may have pharmacological and medicinal applications. Fungi were cultured from marine brown algae and identified using multiple target genes to confirm phylogenetic placement. These target genes included the internal transcribed spacer (ITS), the nuclear large subunit (LSU), and the β-tubulin region. Various biological activities of marine-derived fungi were evaluated, including their antifungal, antioxidant and cellulolytic enzyme activities. As a result, a total of 50 fungi was isolated from the brown algae *Sargassum* sp. Among the 50 isolated fungi, *Corollospora angusta* was the dominant species in this study. The genus *Arthrinium* showed a relatively strong antifungal activity to all of the target plant pathogenic fungi. In particular, *Arthrinium saccharicola* KUC21221 showed high radical scavenging activity and the highest activities in terms of filter paper units (0.39 U/mL), endoglucanase activity (0.38 U/mL), and β-glucosidase activity (1.04 U/mL).

## 1. Introduction

The marine environment is extremely complex and contains a broad spectrum of fungal diversity. Although a variety of new marine-derived fungal genera have been identified and evaluated [1], numerous marine fungi are identical to terrestrial fungi, e.g., *Aspergillus* spp., *Cephalosporium* spp., *Fusarium* spp., or *Penicillium* spp. [2,3]. Marine-derived fungi have been discovered in various locations such as sediment, sponges, microalgae, fish, the deep sea and mangrove wood [4]. However, high-resolution taxonomic identification of marine fungi is still lacking because the majority of studies have only been resolved to the genus level [5,6,7]. Because species within a genus can differ highly in their biological activities, it is necessary to assess DNA-based information at the finest resolution. Thus, the fungal internal transcribed spacer (ITS) is commonly used to identify fungi [8,9] but is unable to classify some taxa at the species level [10,11]. To remedy this deficiency in accuracy and show high resolution in early diverging lineages, a multi-gene phylogenetic analysis was utilized [10,12]. Numerous marine-derived fungi were investigated through multi-gene phylogeny [3,13].

In addition, immense efforts have been made to investigate marine-derived compounds. Unique and stressful marine habitats have profound effects on fungal biological activity. Many marine-derived fungal biological activities have been detected, including antifungal, antioxidant, cytotoxic, antitumor, or anti-inflammatory activities [14,15]. Cephalosporin C in *Acremonium chrysogenum* was the first bioactive compound found from a marine fungus [16]. Since these discoveries, investigations into natural products and the biological activities of marine-derived fungi have been increasing. Several novel marine natural products were isolated and described. The genus *Aspergillus* from the marine habitat is well known as a producer of new compounds. Three new phenolic bisabolance-type sesquiterpenoids, meroterpenoids, and aspergillides A–C were isolated from marine-derived *Aspergillus* sp. [17], *A. flavipes* [18], and *A. ostianus* [19], respectively. In particular, avrainvillamide was isolated from marine-derived *Aspergillus* sp. CNC358 and demonstrated chemotherapeutic properties; it has also shown licensed *in vivo* activity in preclinical models [20]. Recently, components that were isolated from marine-derived fungi were used in the cosmetic industry. The marine-derived fungal carotenoid and kojic acid are used as ingredients in natural pigments and skin-whitening products [21]. These attractive applications encourage the bio-mining of marine environments. Because cellulosic compounds are in high abundance in marine systems, marine fungi are candidates for the production of novel cellulosic compounds. Additionally, marine fungi are great cellulolytic enzyme production candidates, considering the abundance of cellulosic materials in the marine environment [22,23]. Previous studies have reported that marine fungi have cellulase activities that are comparable to those of terrestrial wood-decaying fungi [24,25]. The goal of this study was to evaluate the diversity of fungi collected from the brown algae, *Sargassum* sp., using multigene phylogenetic analysis and to investigate the antifungal, antioxidant, and cellulolytic activities in hopes of finding prospective bioresource agents.

## 2. Results and Discussion

### 2.1. Fungal Phylogeny and Identification

A total of 50 fungal strains were isolated and investigated from the brown algae (Table 1). Two were identified as zygomycetes: *Mucor circinelloides*. The other 48 were identified as ascomycetes. Among isolated fungi, six strains were not identified at the species or genus level because their best matched references from BLAST search were lower than 95% of similarities.

**Figure 1:**
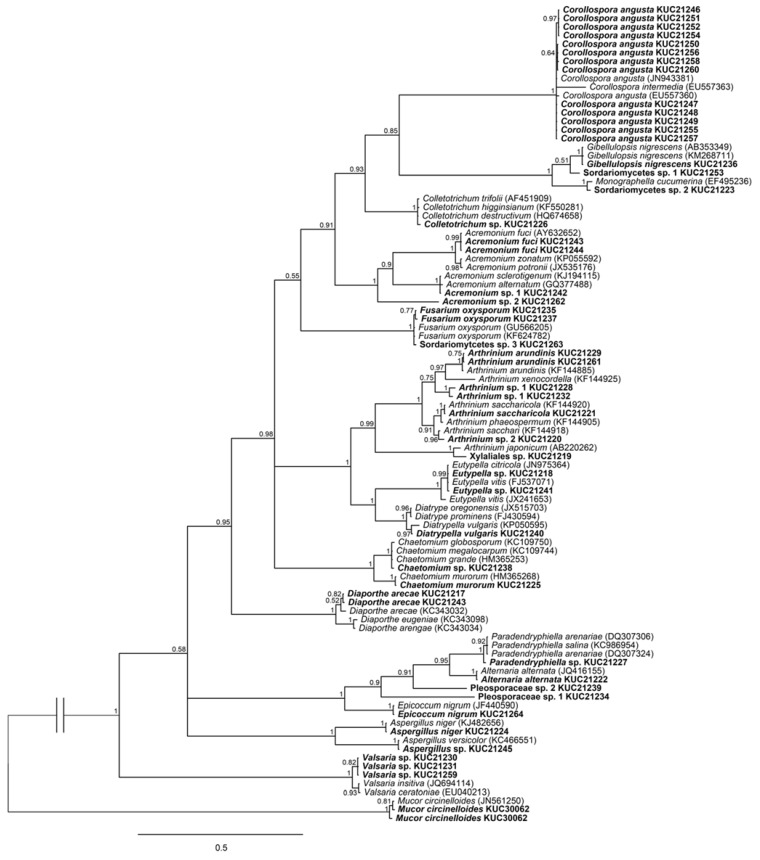
Phylogenetic tree of the fungi isolated from *Sargassum* sp. (in bold) and their allies based on Internal Transcribed Spacer (ITS). Numbers above branches indicate posterior probabilities. The scale bar indicates nucleotide substitutions per position.

In the phylogenetic tree based on ITS (Figure 1), most species were grouped in their own clade. However, five species, *i.e.*, *Acremonium* sp. 1, *Chaetomium* sp., *Colletotrichum* sp., *Eutypella* sp., and *Paradendryphiella* sp., were identified at the genera level because they were grouped in their respective species complex. Six species, *i.e.*, *Acremonium* sp. 2, *Arthrinium* sp. 1, *Arthrinium* sp. 2, Pleosporaceae sp. 1, Pleosporaceae sp. 2, and *Valsaria* sp., were identified at genera or family level due to their best matched sequences from BLAST search had low similarities. They were placed in their own clade without close reference species. Four species, *i.e.*, *Aspergillus* sp., *Sordariomycetes* sp. 2, *Sordariomytcetes* sp. 3, and *Xylaliales* sp., were not identified at species level, despite their own clades with references, because two of the other analyzed regions, LSU and β-tubulin, were not supported by the ITS-based identifications.

**Figure 2:**
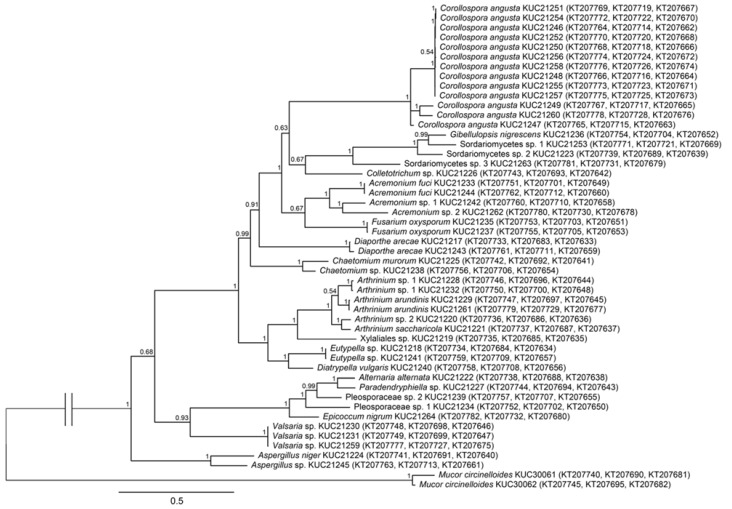
Phylogenetic tree of the fungi isolated from *Sargassum* sp. and their allies based on combined Internal Transcribed Spacer (ITS), nuclear large subunit rDNA (LSU) and β-tubulin sequence alignment. Numbers above branches indicate posterior probabilities. The scale bar indicates nucleotide substitutions per position. Genbank accession numbers of ITS, LSU, and β-tubulin sequences are in parentheses.

The phylogenetic relationship of the ITS-based tree was not clear because some nodes had low posterior probabilities. Thus, phylogenetic tree based on combined dataset from ITS, LSU and β-tubulin sequences was constructed to confirm simultaneous relationship of the isolates (Figure 2). The outline of this tree was similar to the ITS-based tree. Especially, branching pattern of early diverging nodes that classified taxa into class level was agreed with the ITS-based tree. Therefore, the phylogenetic tree based on ITS region could represent their phylogenetic relationships.

**Figure 3:**
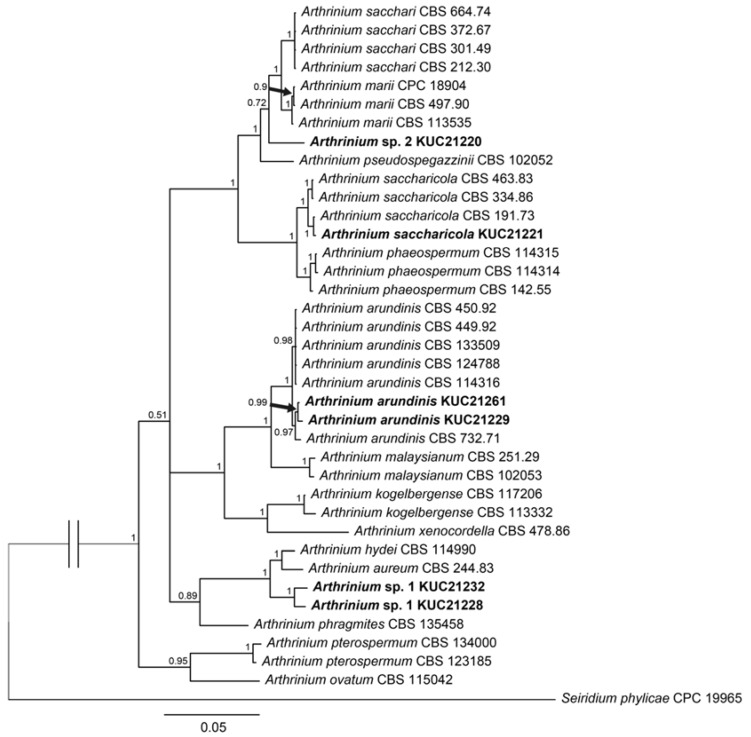
Phylogenetic tree of the *Arthrinium* spp. isolated from *Sargassum* sp. (in bold) and their allies based on combined Internal Transcribed Spacer (ITS), nuclear large subunit rDNA (LSU) and β-tubulin sequence alignment. Numbers above branches indicate posterior probabilities. The scale bar indicates nucleotide substitutions per position.

The most abundant species was *Corollospora angusta* (26%). *C. angusta* has a broad distribution in marine habitats, such as algae and mangroves [26,27,28], but it has not been reported in Korea. It has been suggested that this species is highly associated with its substrate, *Sargassum* sp. In both MEA and PDA media, this species showed a very slow growth rate (less than 1 mm per day). The most diverse genus was *Arthrinium* (12%), which contained four species (Figure 2 and Figure 3). *Arthrinium* is a commonly isolated genus from not only plants and soil but also marine environments [7,11]. In Korea, two *Arthrinium* species, *A. arundinis* and *A. phaeospermum*, have been reported [29,30] but have not been fully described. *A. arundinis* is also known as a plant pathogen of barley [31]. In Korea, *A. arundinis* was isolated from bamboo [30], but marine *A. arundinis* has not been reported. The first *A. saccharicola* was isolated from *Saccharum officinarum* [32]. Although *A. saccharicola* has been reported from various habitats, such as plants, air [11], and seawater [33], it has not been reported in Korea.

**Table 1:** Biological activities of marine fungi isolated from brown algae. ^a^ ABTS, 2,2′-azinobis-(3-ethylbenzothiazoline-6-sulfonic acid); ^b^ DPPH, 2,2-diphenyl-1-picrylhydrazyl; ^c^ FPU, filter paper unit; ^d^ EG, endoglucanase; ^e^ BGL, β-glucosidase; ^f^ BXL, β-xylosidase; ^g^ N.D., not detected.

Fungal Identity	ID	Antifungal Activity IC_50_ (μg/mL)			Antioxidant Activity IC_50_ (μg/mL)		Cellulolytic Enzyme Activity (U/mL)			
		*Botrytis cinerea*	*Collectotrichum gloeosporioides*	*Fusarium oxysporum*	ABTS ^a^ Radical Scavenging Activity	DPPH ^b^ Radical Scavenging Activity	FPU ^c^	EG ^d^	BGL ^e^	BXL ^f^
**Ascomycota**										
**Dothideomycetes**										
*Alternaria alternata*	KUC21222	N.D. ^g^	N.D.	>1000	>1000	N.D.	N.D. ^g^	N.D.	0.07 ± 0	N.D.
*Epicoccum nigrum*	KUC21264	>1000	>1000	N.D.	N.D.	N.D.	0.05 ± 0.09	N.D.	0.05 ± 0.01	0.02 ± 0
*Paradendryphiella* sp.	KUC21227	>1000	N.D.	>1000	N.D.	N.D.	0.15 ± 0	0.03 ± 0.06	0.04 ± 0	N.D.
Pleosporaceae sp. 1	KUC21234	N.D.	>1000	N.D.	N.D.	N.D.	0.1 ± 0.09	N.D.	0.03 ± 0.02	N.D.
Pleosporaceae sp. 2	KUC21239	N.D.	N.D.	>1000	N.D.	N.D.	N.D.	0.03 ± 0.06	0.05 ± 0	N.D.
*Valsaria* sp.	KUC21230	>1000	N.D.	>1000	>1000	N.D.	0.05 ± 0.08	0.04 ± 0.07	0.01 ± 0.02	N.D.
	KUC21231	N.D.	N.D.	N.D.	N.D.	N.D.	N.D.	N.D.	0.03 ± 0.02	N.D.
	KUC21259	N.D.	N.D.	559.0	>1000	N.D.	N.D.	0.08 ± 0.08	0.02 ± 0.03	N.D.
**Eurotiomycetes**										
*Aspergillus niger.*	KUC21224	>1000	>1000	72.5	272.2	>1000	0.15 ± 0	0.04 ± 0.08	0.05 ± 0	0.02 ± 0
*Aspergillus* sp.	KUC21245	>1000	N.D.	>1000	>1000	N.D.	N.D.	0.14 ± 0.02	0.11 ± 0.01	0.02 ± 0
**Sordariomycetes**										
*Acremonium fuci*	KUC21233	N.D.	N.D.	>1000	N.D.	N.D.	N.D.	N.D.	N.D.	N.D.
	KUC21244	523.8	>1000	N.D.	N.D.	N.D.	N.D.	0.03 ± 0.06	0.01 ± 0.02	N.D.
*Acremonium* sp. 1	KUC21242	N.D.	>1000	N.D.	N.D.	N.D.	N.D.	N.D.	N.D.	N.D.
*Acremonium* sp. 2	KUC21262	N.D.	N.D.	670.7	>1000	N.D.	0.1 ± 0.09	0.11 ± 0	0.07 ± 0.01	N.D.
*Arthrinium arundinis*	KUC21229	N.D.	N.D.	193.1	N.D.	N.D.	0.1 ± 0.09	0.17 ± 0.04	0.05 ± 0	N.D.
	KUC21261	>1000	590.3	225.9	N.D.	N.D.	0.1 ± 0.09	0.18 ± 0.07	0.04 ± 0	N.D.
*Arthrinium saccharicola*	KUC21221	210.5	N.D.	>1000	46	88.4	0.39 ± 0.13	0.38 ± 0	1.04 ± 0.03	0.02 ± 0
*Arthrinium* sp. 1	KUC21228	574.8	N.D.	391.8	N.D.	N.D.	0.15 ± 0	0.26 ± 0.04	0.1 ± 0.01	0.02 ± 0
	KUC21232	172.8	>1000	139.9	N.D.	N.D.	0.15 ± 0	0.14 ± 0.02	0.06 ± 0	0.01 ± 0.01
*Arthrinium* sp. 2	KUC21220	179.9	>1000	>1000	>1000	>1000	0.15 ± 0	0.14 ± 0.02	0.16 ± 0.04	0.02 ± 0
*Chaetomium murorum*	KUC21225	N.D.	N.D.	>1000	N.D.	N.D.	N.D.	N.D.	0.04 ± 0	N.D.
*Chaetomium* sp.	KUC21238	N.D.	N.D.	>1000	N.D.	N.D.	N.D.	0.07 ± 0.06	0.04 ± 0	N.D.
*Colletotrichum* sp.	KUC21226	>1000	N.D.	>1000	N.D.	N.D.	0.15 ± 0	0.13 ± 0.01	0.04 ± 0	0.01 ± 0.01
*Corollospora angusta*	KUC21246	>1000	N.D.	N.D.	N.D.	N.D.	N.D.	N.D.	N.D.	N.D.
	KUC21247	N.D.	N.D.	>1000	N.D.	N.D.	N.D.	N.D.	N.D.	N.D.
	KUC21248	>1000	N.D.	>1000	N.D.	N.D.	N.D.	N.D.	N.D.	N.D.
	KUC21249	N.D.	N.D.	N.D.	N.D.	N.D.	0.05 ± 0.09	N.D.	N.D.	N.D.
	KUC21250	N.D.	N.D.	N.D.	N.D.	N.D.	0.1 ± 0.09	N.D.	N.D.	N.D.
	KUC21251	N.D.	N.D.	N.D.	N.D.	N.D.	0.1 ± 0.09	N.D.	N.D.	N.D.
	KUC21252	N.D.	N.D.	N.D.	N.D.	N.D.	N.D.	N.D.	N.D.	N.D.
	KUC21254	>1000	N.D.	N.D.	N.D.	N.D.	N.D.	N.D.	N.D.	N.D.
	KUC21255	N.D.	N.D.	N.D.	N.D.	N.D.	N.D.	N.D.	0.04 ± 0	N.D.
	KUC21256	N.D.	>1000	>1000	N.D.	N.D.	N.D.	N.D.	0.01 ± 0.02	N.D.
	KUC21257	>1000	>1000	N.D.	N.D.	N.D.	N.D.	N.D.	N.D.	N.D.
	KUC21258	>1000	>1000	N.D.	N.D.	N.D.	N.D.	N.D.	N.D.	N.D.
	KUC21260	>1000	N.D.	>1000	N.D.	N.D.	N.D.	N.D.	N.D.	N.D.
*Diaporthe arecae*	KUC21217	N.D.	N.D.	N.D.	N.D.	N.D.	0.16 ± 0.01	0.14 ± 0.03	0.29 ± 0.08	0.01 ± 0.01
	KUC21243	>1000	N.D.	N.D.	N.D.	N.D.	0.1 ± 0.09	0.13 ± 0.02	0.13 ± 0.07	0.01 ± 0.01
*Diatrypella vulgaris*	KUC21240	N.D.	N.D.	758	N.D.	N.D.	N.D.	N.D.	N.D.	N.D.
*Eutypella* sp.	KUC21218	>1000	N.D.	N.D.	N.D.	N.D.	0.05 ± 0.09	0.14 ± 0.01	0.18 ± 0.04	0.02 ± 0
	KUC21241	N.D.	>1000	>1000	N.D.	N.D.	N.D.	0.16 ± 0.05	0.17 ± 0.09	0.01 ± 0.01
*Fusarium oxysporum*	KUC21235	N.D.	N.D.	>1000	N.D.	N.D.	0.15 ± 0	N.D.	0.04 ± 0	0.01 ± 0.01
	KUC21237	N.D.	N.D.	>1000	N.D.	N.D.	N.D.	N.D.	0.04 ± 0	N.D.
*Gibellulopsis nigrescens*	KUC21236	N.D.	N.D.	>1000	N.D.	N.D.	0.05 ± 0.09	0.04 ± 0.06	0.04 ± 0	N.D.
*Sordariomycetes* sp. 1	KUC21253	>1000	N.D.	N.D.	N.D.	N.D.	N.D.	N.D.	N.D.	N.D.
*Sordariomycetes* sp. 2	KUC21223	>1000	N.D.	N.D.	N.D.	N.D.	N.D.	N.D.	N.D.	N.D.
*Sordariomycetes* sp. 3	KUC21263	N.D.	N.D.	>1000	N.D.	N.D.	N.D.	N.D.	0.04 ± 0	N.D.
*Xylariales* sp.	KUC21219	>1000	>1000	97.9	>1000	>1000	0.05 ± 0.09	N.D.	0.05 ± 0.01	0.01 ± 0.01
**Zygomycota**										
**Mucoromyciotina**										
*Mucor circinelloides*	KUC30061	>1000	N.D.	>1000	>1000	N.D.	0.05 ± 0.09	N.D.	0.13 ± 0.03	0.01 ± 0.01
	KUC30062	N.D.	N.D.	N.D.	N.D.	N.D.	0.15 ± 0	0.04 ± 0.06	0.05 ± 0.01	N.D.

In our phylogenetic tree of *Arthrinium* spp. (Figure 3), each *Arthrinium* species was classified at the species level with high posterior probability (p.p.). *A. arundinis* KUC21261 and KUC21229 were placed in a monophyletic clade of *A. arundinis* with a high p.p. (1.0). *A. saccharicola* KUC21221 was grouped with reference *A. saccharicola* with a high p.p. (1.0). Conversely, *Arthrinium* sp. 1 KUC21228, KUC21232, and *Arthrinium* sp. 2 KUC21220 did not join the clade with reference *Arthrinium* but instead had their own clades. They also demonstrated low similarities with best matched references from BLAST search. We suggest that these unidentified *Arthrinium* are new species candidates. Further studies with detailed morphological analyses are required to prove the novelty of these species.

*Acremonium* was the third largest genus in this study (8%). It is a cosmopolitan genus that is commonly isolated from soil, wood [34,35], and seaweed [7,36]. *Aspergillus* spp. is a common saprophyte of various substrates [35], and two species were isolated. Some *Aspergillus* species are halophilic and are commonly observed in marine environments [7,37]. *A. niger* is common in various environments, including seawater [38,39]. In addition, *Fusarium oxysporum*, a well-known plant pathogen [40], is a commonly isolated soil fungus worldwide [35], and has been found in marine environments [41].

*Mucor circinelloides* was the solitary zygomycetous species found in this study. The genus, *Mucor* has been reported to occur in a variety of seaweed [7]. This saprophytic species is commonly isolated from various environments [35], including seawater [42], and has been utilized in the production of fermented food [43,44] and in biotechnology applications to produce lipids [45] and ethanol [46].

For marine fungi which were not identifiable at the species or genus level, further studies with morphological approaches are required. Certainly, identified fungal diversity data can help further studies on marine fungal diversities.

### 2.2. Biological Activities of the Strains

#### 2.2.1. Antifungal Activity and Antioxidant Activity

The antifungal activity of extracts from each strain were examined against three species, *Fusarium oxysporum* KUC20267, *Botrytis cinerea* KUC21265, and *Collectotrichum gloeosporioides* KUC21266 (Table 1) in triplicate. At the same dose, the most inhibited phytopathogenic fungus was *F. oxysporum*. Five strains showed remarkable activities against *F. oxysporum*: *Xylariales* sp. KUC21219, *Aspergillus niger* KUC21224, *A. arundinis* KUC21229, KUC21261, and *Arthninium* sp. 1 KUC21232. And *B. cinerea*, a pathogen of the grape berry and apples, was inhibited by four strains: *A. saccharicola* KUC21221, *Arthrinium* sp. 1 KUC21228, *Arthrinium* sp. KUC21232 and *Arthrinium* sp. 2 KUC21220. In contrast, *C. gloeosporioides* was weakly inhibited by marine-derived fungal extracts in this study. Plant pathogenic fungi are the main infection sources of damage during pre- or post-harvest in the agricultural industry.

There are several studies on the biological control of marine-derived compounds against phytopathogenic fungi [14]. Aqueous and ethanolic extracts from marine algae were shown to inhibit *B. cinerea* and *Phytophthora cinnamon* [47].

In this study, the genus *Arthrinium* showed potential antifungal activity to target fungi. To date, over 20 compounds have been isolated and studied from the genus *Arthrinium*. In particular, the origins of some compounds were marine habitats. Four new compounds, arthrinins A–C and arthrinins D were isolated from the sponge-derived *Arthrinium* sp. [48]. Additionally, (−)-hexylitaconic acid was isolated from sponge-derived *Arthrinium* sp. [49]. Three new chemical compounds, *i.e.*, myrocin D, libertellenone E, and libertellenone F, were isolated from the marine fungus *A. sacchari* [50]. Plant-derived *Arthrinium phaeospermum* produced the antifungal compound arthrinic acid [51]. Biotic pesticide antifungal compounds can be useful in the control of infections caused by phytopathogenic fungi. The results of this study demonstrate the possibility of biological control of marine-derived fungal extracts.

#### 2.2.2. Antioxidant Activity

In this study, 50 marine-derived fungal extracts were screened for their antioxidant capacity. The antioxidant activity was measured by the 2,2′-azinobis-(3-ethylbenzothiazoline-6-sulfonic acid) (ABTS) and 2,2-diphenyl-1-picrylhydrazyl (DPPH) radical scavenging activity. Among the fungal extracts, *Arthrinium saccharicola* KUC21221 and *Aspergillus niger* KUC21224 showed strong ABTS radical scavenging. *A. saccharicola* KUC21220 exhibited the highest DPPH radical scavenging activity, although *A. niger* KUC21224, *Arthrinium* sp. 2 KUC21220 and *Xylariales* sp. KUC21219 also exhibited activities. To the best of our knowledge, this is the first report of *A. saccharicola* activity on radical scavenging. Additionally, *Diaporthe arecae*, which is generally considered a plant pathogenic fungus, exhibited an antioxidant effect. Numerous marine filamentous fungi can produce antioxidant compounds and play an important role in the capture of free radicals. Excess free radicals or reactive oxygen species are harmful to human life [52]. Other studies have shown that various marine-derived fungal extracts are potent radical scavengers [53,54]. These efficient antioxidant compounds can be applied to the cosmetic industry. Kojic acid, a tyrosinase inhibitor, was screened through 600 marine-derived fungi and has been used as a cosmetic agent [21]. As an additional experiment, the radical scavenging activities of the extracts of cellulase-inducing media were investigated. However, there were no positive results among the extracts, although many fungal strains, such as *A. saccharicola*, showed high radical scavenging activity when cultivated on PDA (Table 1). It is believed that fungi are poor producers of antioxidants in cellulase-inducing media. This influence of culture media, such as the carbon source, on the secretion of secondary metabolites by fungi has been previously identified [55,56].

#### 2.2.3. Cellulolytic Enzymes

To investigate the specific cellulolytic enzyme activities of the isolated fungi, the filter paper unit (FPU), endoglucanase (EG), β-glucosidase (BGL) and β-xylosidase (BXL) activities of all fungal strains were measured. FPU indicates the total cellulolytic enzyme activity. EG creates a free chain-end by attacking the non-crystalline regions of the cellulose fiber. BGL acts on β-1,4 bonds linking two glucose or glucose-substituted molecules, and BXL catalyzes the hydrolysis of 1,4-β-d-xylans [57]. Among the fungal isolates, *Arthrinium saccharicola* KUC21221 showed the highest activities in FPU (0.39 U/mL), EG (0.38 U/mL), and BGL (1.04 U/mL), and it showed a remarkable radical scavenging activity in this study (Table 1). These activities were comparable to the reported values of other wood-decaying fungi (Table 2). The FPU activity of *A. saccharicola* KUC21221 was also higher than that of *Penicillium echinulatum* (0.27 U/mL) [58]. It was expected that *A. saccharicola* KUC21221 would have a great saccharification ability because the FPU is closely related to the saccharification yield. According to a previous study, *Hypoxylon oceanicum* showed the highest EG activity among the five fungi isolated from mangroves [59]. However, *A. saccharicola* KUC21221 showed much higher activity in EG than did *H. oceanicum* (0.04 U/mL), and it exhibited higher BGL activity than did *Aspergillus niger* (1.02 U/mL) [60]. High BGL activity is important because BGL minimizes product inhibition and transglycosylation and promotes the production of cellulase inducer compounds [61]. Considering that *A. saccharicola* KUC21221 was derived from the marine environment, it was expected that it may secrete more enzyme when cultured in media with a proper amount of salinity [62]. Conversely, no strain showed a highly elevated BXL activity. Because all of the fungal strains were isolated from brown algae, low BXL activities could occur because the hemicellulose content is low in brown algae [63]. Our results support these fungal characteristics and further demonstrate the untapped potential of novel fungal lineages and their potentially useful metabolites in marine systems.

**Table 2:** Quantitative comparison of enzyme activities of *Arthrinium saccharicola* KUC21221 with other terrestrial fungi. ^a^ FPU, filter paper unit; ^b^ EG, endoglucanase; ^c^ BGL, β-glucosidase.

Enzyme	Strains	Substrate	Cultivation Conditions	Activity (U/mL)	References
FPU ^a^	*A. saccharicola* KUC 21221	Cellulose	Shake flask, 25 °C, 168 h	0.39	This study
	*Penicillium echinulatum*	Cellulose	Stir fermenter, 37 °C, 192 h	0.27	[56]
EG ^b^	*A. saccharicola* KUC 21221	Cellulose	Shake flask, 25 °C, 168 h	0.39	This study
	*Hypoxylon oceanicum*	Cellulose + Filter paper	Shake flask, 25 °C, 360 h	0.04	[57]
BGL ^c^	*A. saccharicola* KUC 21221	Cellulose	Shake flask, 25 °C, 168 h	1.04	This study
	*Aspergillus niger*	Cellulose	Shake flask, 28 °C, 168 h	1.02	[58]

## 3. Experimental Section

### 3.1. Sampling and Isolation of Fungi

Marine brown algae, *Sargassum* sp., were collected from Hyeopjae beach in Jeju, Korea. The samples were washed thoroughly with distilled water two times and cut into approximately 0.5 cm × 0.5 cm squares using a sterile surgical blade. Small segments were treated with 70% ethanol for 60 s and then washed in sterile distilled water for 10 s [5,64]. The segments were placed on malt extract agar (MEA) with 0.01% streptomycin and ampicillin to prevent bacterial growth. The grown fungi from the segments were transferred to MEA periodically and incubated at 25 °C.

### 3.2. DNA Extraction, PCR and Identification

Genomic DNA was extracted from fungal cultures using the AccuPrep Genomic DNA Extraction Kit (Bioneer, Seoul, Korea) according to the manufacturer’s protocol. PCR reactions were carried out using the AccuPower PCR Premix Kit (Bioneer, Seoul, Korea) with the primers for the internal transcribed spacer (ITS) being ITS1F (5′-CTT GGT CAT TTA GAG GAA GTA A-3′) [65] and ITS4 (5′-TCC TCC GCT TAT TGA TAT GC-3′) [8], for nuclear large subunit rDNA (LSU) LR0R (5′-ACC CGC TGA ACT TAA GC-3′)/LR5 (5′-TCC TGA GGG AAA CTT CG-3′) or LR0R/LR7 (5′-TAC TAC CAC CAA GAT CT-3′) [66], and for β-tubulin T10 (5′-ACG ATA GGT TCA CCT CCA GAC-3′) /T2 (5′-TAG TGA CCC TTG GCC CAG TTG-3′) [67] or Bt2a (5′-GGT AAC CAA ATC GGT GCT GCT TTC-3′)/Bt2b (5′-ACC CTC AGT GTA GTG ACC CTT GGC-3′) [68]. PCR amplification conditions for ITS and LSU were under the following temperature cycling parameters: 95 °C for 4 min, followed by 30 cycles of 95 °C for 30 s, 55 °C for 30 s, and 72 °C for 30 s. An elongation step of 72 °C for 5 min was performed at the end. For β-tubulin, the conditions were as follows: 95 °C for 5 min, followed by 30 cycles of 95 °C for 35 s, 55 °C for 50 s, and 72 °C for 2 min; an elongation step was performed at 72 °C for 7 min. DNA sequencing was performed using Sanger method with 3730xl DNA Analyzer (Life technology, Carlsbad, CA, USA) by Macrogen (Seoul, Korea). The sequences obtained in this study were deposited and the GenBank accession numbers are in Figure 2. The obtained DNA sequences were proofread, and a BLAST search was performed (http://blast.ncbi.nlm.nih.gov/Blast.cgi).

### 3.3. Phylogenetic Analysis

The obtained sequences were aligned using MAFFT 7.130 [69] and modified manually using MacClade 4.08 [70]. The ITS, LSU and β-tubulin datasets contained 50 taxa and 778, 1005 and 657 nucleotide characters, respectively. They were respectively tested by MrModeltest 2.3 with default options using the AIC criteria [71]. The GTR+I+G model was chosen under the AIC criteria for all datasets. Three datasets were combined and the selected model was applied. Bayesian analysis was performed using MrBayes 3.2.1 [72]. Two runs with 1,000,000 generations were performed, and every 100th generation was sampled. Among them, the first 25% of the trees was eliminated, and the last 75% was used. A 50% majority-rule consensus tree was constructed, and tree reliability was confirmed by posterior probability.

The phylogenetic tree based on ITS was constructed to prove the identity of each fungus. The ITS sequences were aligned using MAFFT 7.130 with reference sequences obtained from GenBank using a BLAST search. The dataset has 95 taxa and 810 nucleotide characters. Phylogenetic analysis was performed using the described method. The GTR+I+G model was selected under the AIC criteria.

To confirm the identification of *Arthrinium* spp., which shows strong bioactivity, an additional phylogenetic tree was constructed with reference sequences from Crous and Groenewald [11]. Phylogenetic analysis was performed as described in this study. The ITS, LSU and β-tubulin datasets contained 38 taxa and 643, 893 and 560 nucleotide characters, respectively. The SYM+I+G model was selected for the ITS dataset, and the GTR+I+G model was chosen for the last two datasets.

### 3.4. Biological Activity

#### 3.4.1. Preparation of Extracts

A total of 50 fungal species were cultivated on 50 mL of potato dextrose agar (PDA; Difco, Detroit, MI, USA) at 25 °C for 7 days in the dark. After the incubation periods, the solid culture was extracted with 200 mL of methanol (SK chemicals, Ulsan, Korea) for a day. The harvested MeOH solution was filtered through Whatman No. 1 filter paper. The solvents were evaporated to dryness under a vacuum at 37 °C, and 4 °C was used during the cooling circulation to obtain effective yields. The condensed residues were re-dissolved in ethyl acetate and distilled water (1:1). Then, the partitioned ethyl acetate fraction was evaporated. The extracts were solubilized in dimethyl sulfoxide (DMSO; Junsei, Tokyo, Japan) to a final concentration of 10 mg/mL and stored at 4 °C before testing [2].

#### 3.4.2. Antifungal Activity

The capability of marine-derived fungi to control three plant pathogenic fungi was evaluated. *Fusarium oxysporum* KUC20267, *Botrytis cinerea* KUC21265, and *Collectotrichum gloeosporioides* KUC21266 were tested as the target fungi. Each well of a 96-well microplate contained 2 μL of fungal extracts, 20 μL of spore suspension, and 178 μL of media [73]. The plant pathogenic fungi spore suspension was adjusted to 4 × 10^6^ per mL. The absorbance was measured every 24 h at 595 nm.

#### 3.4.3. Antioxidant Activity

##### ABTS Radical-Scavenging Assay

The 2,2′-azinobis-(3-ethylbenzothiazoline-6-sulfonic acid) (ABTS) (Sigma-Aldrich Inc., St. Louis, MO, USA) radical-scavenging activity was measured as described by Roberta *et al.* (1999) [47]. ABTS was dissolved in phosphate-buffered saline (PBS, pH 7.4) to 7 mM, and the ABTS radical cation was produced by adding potassium persulfate at a final concentration of 2.45 mM. The mixture was incubated for 24 h at RT in the dark, and the resultant ABTS radical solution was diluted to an absorbance of 0.70 (±0.02) at 734 nm. A 990 μL aliquot of the prepared ABTS radical solution was reacted with 10 μL of the extracts (10 mg/mL) in a cuvette. After 6 min, the absorbance at 734 nm was measured using a spectrophotometer. Trolox was used as the antioxidant positive control solution.

##### DPPH Radical-Scavenging Assay

The 2,2-diphenyl-1-picrylhydrazyl (DPPH) (Sigma-Aldrich Inc., St. Louis, MO, USA) radical scavenging activity of the samples was analyzed according to Fukumoto *et al.* [4]. The DPPH solution was prepared in methanol (80%) at 150 μM. For analysis in the 96-well plate, we added 22 μL of the extracts of the sample (10 mg/mL) and 200 μL of the DPPH solution to each well and then incubated the plate at room temperature for 30 min. The absorbance at 520 nm was measured using a microplate reader. A control consisted of 22 μL of methanol instead of the sample extracts. Trolox was used as the antioxidant standard.

#### 3.4.4. Cellulolytic Enzyme

##### Enzyme Preparation

All fungal isolates were inoculated on solid medium containing 2% (w/v) malt extract (Bacto, Sparks, MD, USA) and 1.5% agar powder (Showa, Tokyo, Japan). The strains were grown for seven days, and two agar plugs of each strain were used for the inoculums. To prepare the fungal enzyme, the fungi were cultivated in 50 mL conical tubes that contained 10 mL Mandels’ medium (0.3 g of urea, 1.4 g of KH_2_PO_4_, 2.0 g of (NH_4_)_2_SO_4_, 0.3 g of CaCl_2_, 0.3 g of MgSO_4_, 0.25 g of yeast extract, 0.75 g of peptone, 5 mg of FeSO_4_·7H_2_O, 36 mg of COCl_2_·6H_2_O, 1.8 mg of MnSO_4_·H_2_O, and 2.5 mg of ZnSO_4_·7H_2_O per liter of distilled water) with 1% cellulose as the sole carbon source [74]. Fungal cultures were incubated aerobically for 1 week at 25 °C on a rotary shaker at 150 rpm in the dark. The cultures were prepared in triplicate, and the mean values are presented. After cultivation, the samples were centrifuged at 4000 rpm for 25 min at 4 °C, and the supernatant was extracted and filtered through a 0.45 μm filter (Minisart, Sartorius, Göttingen, Germany) to determine the enzymatic activities of the crude enzyme solutions.

##### Enzyme Assays

The FPU activities were measured using a 60 μL filter paper assay (FPA) method [75]. Briefly, the reaction mixtures were prepared in a total volume of 60 μL consisting of 20 μL of the enzyme solutions and 40 μL of 50 mM citrate buffer (pH 4.8) in a 0.2 mL PCR tube (Axygen, cat. No. 321-10-051); the substrate was a 7 mm diameter Whatman No. 1 filter paper disk. After one hour of the reaction at 50 °C, 120 μL of dinitrosalicylic acid (DNS) was added. The tubes were boiled and cooled for 5 min each. Thirty-six microliters of the reaction mixtures were transferred to a 96-well tissue microplate (Falcon, cat. No. 353072) with 160 μL of distilled water, and the absorbance was measured at 540 nm. The absorbance was used to calculate the FPU using glucose as the standard. One FPU was defined as 1 mM of glucose equivalent released per minute. The EG activities were assayed according to standard procedures [76,77]. The procedure was similar to the above FPA method. Briefly, the reaction mixtures were prepared in a total volume of 50 μL consisting of 25 μL of enzyme solutions and 25 μL of 2% CMC in 50 mM citrate buffer (pH 4.8) in a 0.2 mL PCR tube. After one hour of the reaction at 50 °C, 150 μL of the DNS reagent was added. The following procedures were the same as the above FPA method. The tubes were boiled and cooled for 5 min each. Thirty-six microliters of the reaction mixtures was transferred to flat-bottom 96-well tissue culture plates with 160 μL of distilled water, and the absorbance was measured at 540 nm. The absorbance was used to calculate the EG activity using glucose as the standard. One unit of EG activity was defined as 1 mM of glucose equivalent released per minute. The activities of BGL and BXL were determined according to the method of Valásková and Baldrian [78]. These activities were determined by measuring the concentration of *p*-nitrophenyl (pNP) released by *p*-nitrophenyl glucosidase (pNPG) or *p*-nitrophenyl xylosidase (pNPX). Briefly, reaction mixtures consisting of 20 μL of the enzyme solutions, 20 μL of 10 mM pNPG or pNPX, and 20 μL of 1 M sodium acetate buffer (pH 5.0) were incubated for 5 and 10 min, respectively, at 50 °C. The reaction was stopped by adding 20 μL of 2 M Na_2_CO_3_, and the absorbance was measured at 405 nm. One unit of BGL or BXL activity was defined as the amount of enzyme that released 1 mM of pNP per minute [79].

### 3.5. Statistical Analysis

Inhibitory concentration (IC_50_) values were calculated from nonlinear regression analysis using the SAS software (version 9.2 by SAS Institute Inc., Cary, NC, USA).

## 4. Conclusions

In conclusion, this study provided reliable DNA information for marine-derived fungi and their biological activities. A total of 50 fungal strains were obtained from the brown algae *Sargassum* sp. Based on the BLAST search with ITS, LSU, and the β-tubulin region, a phylogenetic analysis was performed to verify the identification. Among the collected fungi, *Corollospora angusta* was the dominant species, and the most diverse genus was *Arthrinium*. In addition, *Arthrinium* spp. showed relatively strong biological activities for antifungal, antioxidant, and cellulolytic activity. The potential bioactive compounds will be isolated and purified in the near future.
